# Purification and characterization of a novel phloretin-2′-*O*-glycosyltransferase favoring phloridzin biosynthesis

**DOI:** 10.1038/srep35274

**Published:** 2016-10-12

**Authors:** Tingjing Zhang, Jianqiang Liang, Panxue Wang, Ying Xu, Yutang Wang, Xinyuan Wei, Mingtao Fan

**Affiliations:** 1 College of Food Science and Engineering, Northwest A&F University, Yang ling, Shaanxi, 712100, China; 2 College of Life Sciences, Northwest A&F University, Yangling, Shaanxi, 712100, China; 3 Department of Food Science, University of Massachusetts, Amherst, MA01003, USA; 4 College of Life Science and Engineering, Shaanxi University of Science & Technology, Xi’an, Shaanxi, 710021, China

## Abstract

Phloretin-2′-*O*-glycosyltransferase (P2′GT) catalyzes the last glycosylation step in the biosynthesis of phloridzin that contributes to the flavor, color and health benefits of apples and processed apple products. In this work, a novel P2′GT of *Malus x domestica (Md*P2′GT) with a specific activity of 46.82 μkat/Kg protein toward phloretin and uridine diphosphate glucose (UDPG) at an optimal temperature of 30 °C and pH 8.0 was purified from the engineered *Pichia pastoris* broth to homogeneity by anion exchange chromatography, His-Trap affinity chromatography and gel filtration. The purified *Md*P2′GT was low *N*-glycosylated and secreted as a stable dimer with a molecular mass of 70.7 kDa in its native form. Importantly, *Md*P2′GT also exhibited activity towards quercetin and adenosine diphosphate glucose (ADPG), kaempferol and UDPG, quercetin and UDP-galactose, isoliquiritigenin and UDPG, and luteolin and UDPG, producing only one isoquercitrin, astragalin, hyperoside, isoliquiritin, or cynaroside, respectively. This broad spectrum of activities make *Md*P2′GT a promising biocatalyst for the industrial preparation of the corresponding polyphenol glycosides, preferably for their subsequent isolation and purification. Besides, *Md*P2′GT displayed the lowest K_m_ and the highest k_cat_/K_m_ for phloretin and UDPG compared to all previously reported P2′GTs, making *Md*P2′GT favor phloridzin synthesis the most.

Phloretin and its glycosides belong to a subclass of polyphenols within a larger group of plant-based phenolic bioactive natural products. They exhibit a wide variety of beneficial biological activities, such as anti-oxidant^1^, anti-inflammatory^2^, anti-cancer^3^ and anti-diabetic activities^4^. Phloretin, a precursor of phloridzin, exhibits additional pharmacologically biological functions like anti-tumor^5^ and anti-estrogen activities^6^, and inhibition of cardiovascular disease^3^. However, the use of phloretin as a drug and food additive has been limited due to its weak aqueous solubility, chemical and/or biological instability, and low absorbability^1^,^3^,^7^,^8^. Attachment of sugar moieties to phloretin by glycosylation reactions can increase its aqueous solubility and *in vivo* half life, and typically exerts profound direct or indirect effects on its biological activity^1^,^3^,^7^,^9^,^10^.

Phloridzin, a phloretin glycoside, is biosynthesized via a glycosylation reaction towards phloretin and UDPG catalyzed by phloretin-2′-*O*-glycosyltransferases (P2′GTs)^11^,^12^. Phloridzin is comparable to phloretin in the bioactive properties, but it exhibits greater bioavailability and lower toxicity^1^,^2^,^10^,^13^,^14^ and is thus widely used in diverse commercial products as an alternative to phloretin^7^,^8^. To date, commercial available phloridzin is extracted from non-renewable sources using traditional solvent-based approaches^15^,^16^,^17^,^18^, which restrict its efficient availability. Selective glycosylation of phloretin by P2′GT provides a convenient access to phloridzin through bottom-up synthesis. However, only two P2′GTs, UGT88F1 (accession no. **ACZ44840**) from *M. x domestica* and UGT88F2 (accession no. **ACZ44838**) from *Pyrus communis*, can selectively glycosylate phloretin at the 2′-OH to produce analytical amounts of phlorizin^19^,^20^. Whereas these two P2′GTs appear to exclusively accept phloretin as a glycosyl acceptor. And, the UGT88F2, orthologous to UGT88F1 with 99% homologous sequence identity, can also deglycosylate the formed phloridzin to phloretin and UDP-glucose again, as increasing the incubation time or the concentration of UDP-glucose^7^. These shortcomings limit the availability and usable scope of UGT88F1 and UGT88F2 in the actual production process. Other reported P2′GTs can accept a number of phenylpropanoid substrates, but glycosylation of phloretin by these plant P2′GTs yields mixtures of mono-glycosides^14^,^20^,^21^. A more promiscuous bacterial GT glucosylates phloretin at the 2′-, 4′-, 6′- and 4-OH positions and produces a complex mixture of glycosidic compounds, including two monoglycosides, two diglycosides and one triglycoside^3^. In summary, all reported P2′GTs suffer from a narrow substrate range or low region selectivity, which could reduce economic benefits and complicate the separation and purification of the formed glycosides. Additionally, industrialized societies are moving toward industrial biotechnology, which has proven to be environmentally sound and commercially efficient^22^. This move poses a continuous demand for novel biocatalysts, preferably that can accept a wide range of substrates but do not produce by-products during reactions. Furthermore, P2′GTs belong to the UDP-dependent glycosyltransferases (UGTs) with a supergene family and multiple P2′GTs in apples have been reported to be involved in apple phloridzin biosynthesis^11^,^12^,^19^,^20^,^21^, other isoenzymes might be able to glycosylate phloretin.

Herein, we report a novel recombinant P2′GT that exhibits predominant region specificity for the sugar attachment site and broad-range acceptance for substrates. This P2′GT was produced by an engineered *P. pastoris* GS115 strain harboring a gene (Accession no. **KT444675**) from *M. x domestica (Md*P2′GT). In order to broaden its application range in the actual production process, potential donor substrates and potential acceptor substrates (giving a maximum of 225 combinations) was assayed to evaluate their abilities to act as *Md*P2′GT substrates. The formed glycosides in combinations were sequentially identified by reversed-phase high performance liquid chromatography equipped with a diode array detector (HPLC-DAD), liquid chromatography tandem mass spectrometry (LC-MS/MS), ^1^H Nuclear Magnetic Resonance (^1^H NMR), and ^13^C NMR. Finally, the binding of preferred substrates and the most potent inhibitor with the enzyme active sites was predicted to elucidate key interactions within the *Md*P2′GT/substrate complex. To the best of our knowledge, it is the first time that reports donor substrate and acceptor substrate combinations specificity of P2′GT. Also, our data indicates that *Md*P2′GT is a promising and preferable biocatalyst for efficient glycosylation in natural product synthesis and also simplifies the subsequent isolation and purification of the glycosylated products.

## Results

### Purification of *Md*P2′GT

**Figure 1:**
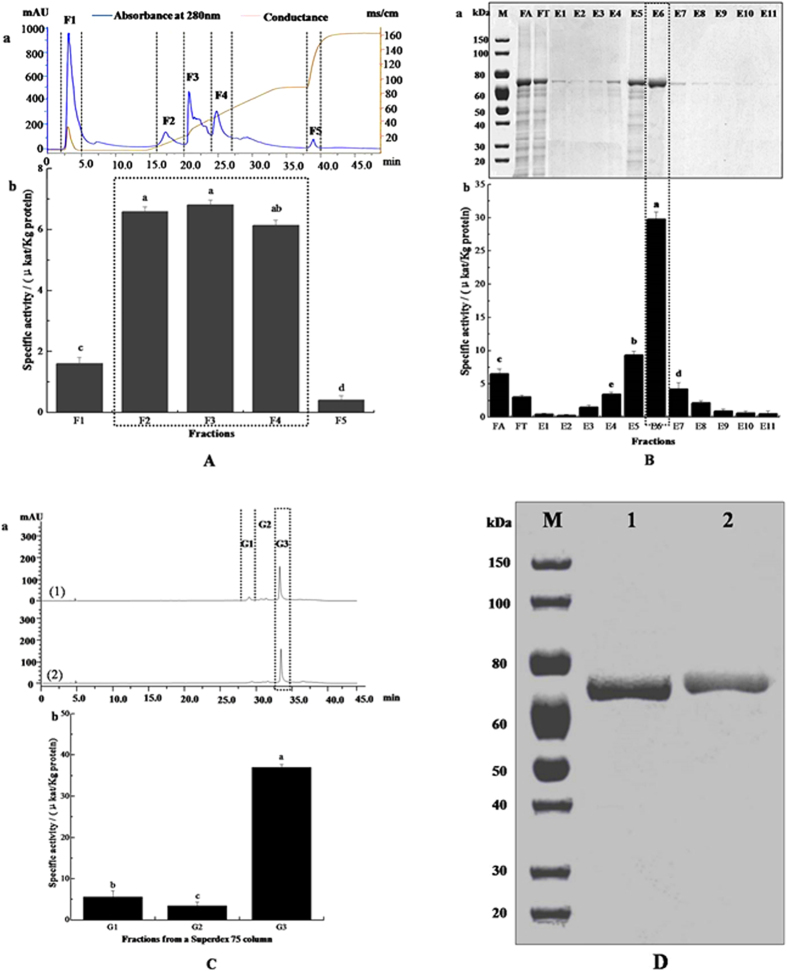
Purification steps of recombinant *Md*P2′GT. (**A**) (a) DEAE ion-exchange chromatography fractions and (b) their corresponding specific activity. (**B**) (a) SDS/PAGE analysis of Ni^2+^ affinity chromatography fractions (FA: mixture of F2-F4 from (**A**); FT: FA flow-through; E1-E11: fractions eluted by buffer C containing 5 mM, 10 mM, 20 mM, 40 mM, 60 mM, 80 mM, 100 mM, 200 mM, 300 mM, 400 mM, and 500 mM imidazole, respectively) and (b) their corresponding specific activity. (**C**) Further purified of E6 in (**B**) by G75 Superdex gel filtration (a(1)elution fractions G1-G3 from E6, a(2)elution fraction G3 purified by gel filtration again) and (b) the corresponding specific activity. (**D**) (1) SDS/PAGE and (2) Western Blot analysis of purified *Md*P2′GT, M: SuperSignal Molecular Weight Protein Ladder.

The three-step purified process used for *Md*P2′GT purification is summarized in Fig. 1 and Table 1. The first purification step (DEAE anion-exchange chromatography) resulted in five enzymatically active fractions (F1–F5) that exhibited significant differences (*P* < 0.05) in specific activity (Fig. 1A), which led to an approximately 59-fold purification with 49.85% yield and a specific activity of 6.48 μkat/Kg protein for *Md*P2′GT (Table 1). His-trap affinity chromatography with 80 mM imidazole as the eluent (E6, Fig. 1B) further increased the purification to 301-fold and the specific activity to 33.13 μkat/Kg protein, with 13.13% yield (Table 1). The gel filtration was finally performed to remove the imidazole in E6, resulting in three fractions (G1-G3) (Fig. 1C(1)). G3, which exhibited the highest specific activity, was loaded again onto a G75 Superdex gel filtration column and yielded a single peak in chromatography (Fig. 1C(2)). Accordingly, approximately 110 mg of purified *Md*P2′GT was obtained from 1 L of engineered *P. pastoris* GS115 culture, the specific activity of which increased from an average of 0.11 μkat/Kg protein in the crude broth to 40.45 μkat/Kg protein, representing a purification of 368-fold with 9.02% recovery. The single peak was further analyzed by sodium dodecyl sulfate polyacrylamide gel electrophoresis (SDS-PAGE) and Western blotting and both yielded a single homogeneous band (Fig. 1D), indicating the purity of *Md*P2′GT was more than 95%. However, the size of purified *Md*P2′GT appeared larger than the theoretical value (53.9 kDa) deduced from the amino acid sequence of mature *Md*P2′GT fused to the His_6_ tag (Fig. S1).

### Molecular weight evaluation and glycosylation analysis

**Figure 2:**
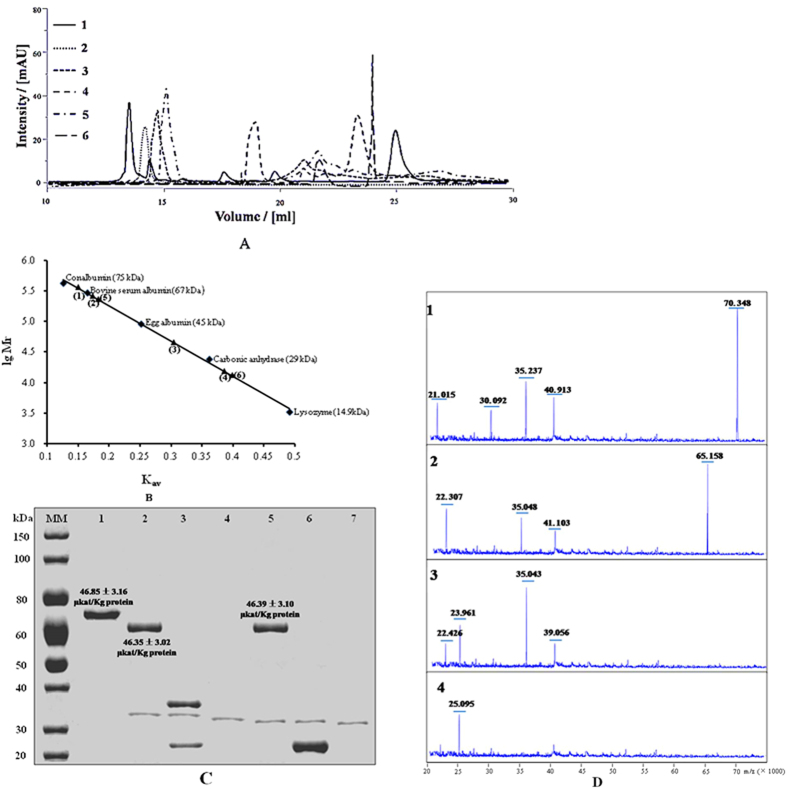
Molecular weight and glycosylation analysis. (**A**) SEC of marker proteins (1, 75 kDa conalbumin, 67 kDa bovine serum albumin, 45 kDa egg albumins, 29 kDa carbonic anhydrase, and 14.9 kDa lysozyme), native *Md*P2′GT (2, calculated MW 70.7 kDa), native (3, calculated MW 65.3 kDa) and denatured (4, calculated MW 35.2 kDa and 24.9 kDa) deglycosylated *Md*P2′GT with PNGase F, and native (5, calculated MW 65 kDa) and denatured (6, calculated MW 24.5 kDa) deglycosylated *Md*P2′GT with Endo H. (**B**) The calibration curve of the marker proteins. (**C**) SDS-PAGE analysis of (1) native *Md*P2′GT, (2) native and (3) denatured deglycosylated *Md*P2′GT with PNGase F, (4) 1 U PNGase F, (5) native and (6) denatured deglycosylation *Md*P2′GT with Endo H, and (7)10 U of Endo H. The specific activity of the native and native deglycosylated *Md*P2′GT under optimal conditions are indicated near the respective lanes. (**D**) MALDI-TOF spectra of (1)the native, (2)the native deglycosylated *Md*P2′GT with PNGase F or Endo H, and denatured deglycosylated *Md*P2′GT with (3)PNGase F or (4)Endo H.

According to the size exclusion chromatography (SEC) and the protein standard curve (Fig. 2A,B), the molecular weight (MW) of purified *Md*P2′GT was approximately 70.7 kDa that is much larger than the theoretical value of 53.9 kDa. To explain this discrepancy, the glycosylation pattern was investigated by deglycosylation with Peptide N-glycosidase F (PNGase F) and Endoglycosidase H (Endo H). Asymmetric tailing of the protein peak in SEC (Fig. 2A2) and the broad signal in SDS-PAGE (Fig. 2C1) [Fig f3][Fig f4]suggested a heterologous glycosylation pattern in *Md*P2′GT. The recombinant *Md*P2′GT was released as a stable dimer with an approximate molecular mass of 65.3 kDa after deglycosylation with PNGase F or Endo H in its native states (Figs 2A3 and 5B,C2,C5). MALDI-TOF mass spectrometric analysis further confirmed that the *Md*P2′GT signal was decreased from 70 kDa to 65 kDa after deglycosylation under native conditions (Fig. 2D1,2). In contrast, under denaturing conditions, *Md*P2′GT was deglycosylated by PNGase F generating two subunits with MWs of 35.2 kDa and 24.9 kDa (Fig. 2). However, when *Md*P2′GT was treated with Endo H under identical conditions, only a monomeric molecular mass of 24.5 kDa was observed (Fig. 2). Hence, approximately 23.8% of the molecular mass is attributable to N-linked carbohydrates, but approximately 30.4% of the glycosylation is resistant to PNGase F treatment under denaturing conditions. Additionally, MALDI-TOF mass spectrometric analysis revealed that asparagine glycosylation was the dominant site for the addition of mannose-containing oligosaccharide and the candidate sites included Asn77, 95, 132, and 195. Finally, the specific activities of native and natively deglycosylated *Md*P2′GT toward phloretin and UDPG were determined. Interestingly, removal of the *Md*P2′GT glycosylation sites resulted in no significant change in specific activity (Fig. 2C). Thus, the purified *MdP*2′GT in this study could be used directly in practice without additional treatments.

### Biochemical characterization of recombinant *Md*P2′GT

**Figure 3 f3:**
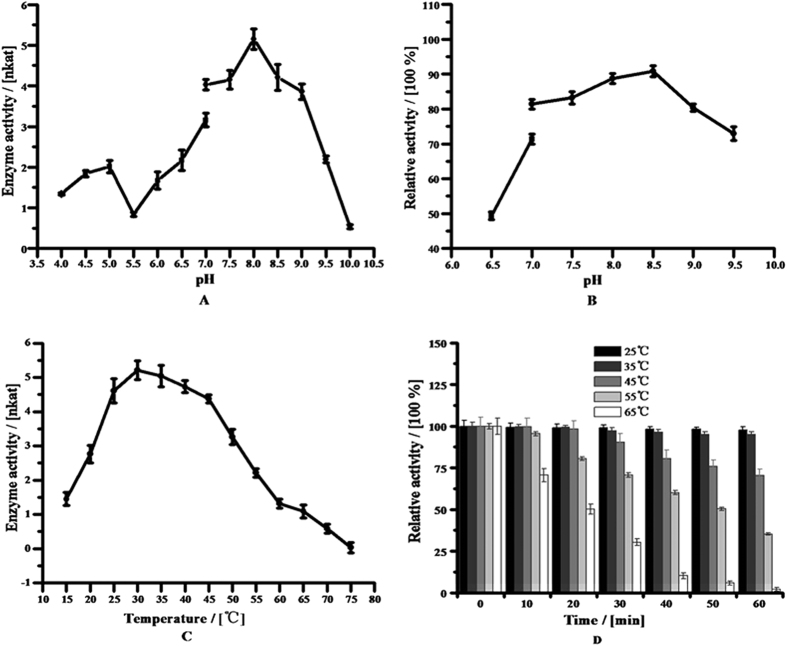
Effects of pH and temperature on enzyme activity and stability. (**A**) Effect and optimum pH. (**B**) pH stability. (**C**) Effect and optimum temperature. (**D**) Temperature stability.

Purified *Md*P2′GT displayed maximal activity at pH 8.0, and up to 80% of its maximal activity remained within the pH range of 7.0–9.0 (Fig. 3A). Moreover, *Md*P2′GT remained stable in the pH range of 7.0–9.0 incubated at 30 °C for 24 h (Fig. 3B). At pH 8.5, the maximal residual activity of *Md*P2′GT was >90% of its original activity, but at pH < 6.5, the residual activity was reduced to <50% (Fig. 3B), indicating that purified *Md*P2′GT preferred alkaline conditions to acidic conditions. Additionally, purified *Md*P2′GT exhibited higher activity and stability in Tris/HCl buffer than in potassium phosphate buffer at pH 7.0 (Fig. 3A,B). Therefore, subsequent biochemical characterizations were performed in Tris/HCl buffer system.

Purified *Md*P2′GT exhibited activity in a broad temperature range from 15 °C to 65 °C, with a maximal activity of 46.18 μkat/Kg protein at 30 °C (Fig. 3C). Its activity was not obviously influenced by incubation at temperatures as high as 45 °C for 20 min but decreased at higher temperatures or longer incubation times (Fig. 3D). Incubated at 45 °C for 60 min, the enzyme retained 70% of its original activity, whereas incubated at 55 °C for 60 min decreased its relative enzyme activity to <40%. Under identical conditions at 65 °C, enzyme activity was only 2% of its original activity (Fig. 3D).

As shown in Table 2, enzyme activity was completely inhibited by 5 mM Co^2+^ and Cu^2+^, partially inhibited by 5 mM Ca^2+^, Mn^2+^, Al^3+^, and Fe^3+^, and negligibly affected by 0.5 mM K^+^, Ni^2+^ and Ba^2+^, while 2 mM Mg^2+^ and 5 mM Fe^2+^ could increase enzyme activity to 130% and 120% of the original activity, respectively. Sodium chloride, used in the purification protocol, had no obvious inhibitory effects even at the concentrations as high as 500 mM. Also, EDTA had no obvious inhibitory effects, suggesting *Md*P2′GT is not a metalloenzyme. However, *Md*P2′GT can be degraded by endogenous *Pichia* proteases and is susceptible to sulfhydryl oxidation, suggesting that adding glycerol, 4-(2-Aminoethyl) benzenesulfonyl fluoride (AEBSF, a protease inhibitor) and β-mercaptoethanol (β-ME) or dithiothreitol (DTT) to the buffers used during purification is essential. No obvious effects of 5% glycerol, 0.5 mM AEBSF, 5 mM β-ME and 5 mM DTT on *Md*P2′GT activity were observed. Due to maximum glycoside product concentrations were restricted by the aqueous solubility of the comparably hydrophobic aglycone acceptors, use of co-solvent conducting the reaction in an aqueous-organic two-phase system can enhance the effective acceptor concentration^7^,^8^. The co-solvents 20% methanol and 20% dimethyl sulfoxide (DMSO) also had no effects on enzyme activity. Whereas the strong inhibition was observed with 1 mM *p*-hydroxymercuribenzoate (PHMB) and 1 mM *N*-Ethylmaleimide (NEM), indicating the presence of an SH group in the active site of *Md*P2′GT. *Md*P2′GT was also completely inhibited by UDP (by-product), strongly inhibited by the other tested product analogs except for uridine at a final concentration of 5 mM. In addition, Higher IC_50_ values indicate lower inhibition, and thus Cu^2+^ was the most effective metal ion inhibitor, UDP was the most potent product inhibitor.

### Substrate specificity of *Md*P2′GT

**Figure 4 f4:**
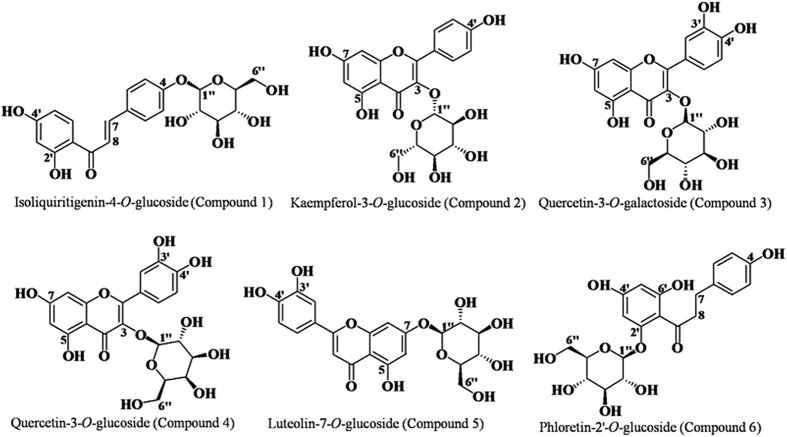
Structural formulas of the glycosides synthesized by *Md*P2′GT.

In this study, a maximum of 225 combinations, resulting from the nine potential glycosyl donor (NDP-sugars) substrates and twenty-five potential glycosyl acceptor (polyphenols) substrates, were evaluated as the substrates for *Md*P2′GT. Only seven combinations were able to act as the enzyme substrates for the production of the corresponding polyphenol glycosides. Phloridzin (phloretin-2′-*O*-glucoside), produced in UDPG/phloretin and ADPG/phloretin combinations, was identified in our previous study (Zhang, *et al*., unpublished data); its chromatography data and structural formula were shown in Table 3 and Fig. 4, respectively. The glycosides formed in the other five combinations were firstly identified by HPLC-DAD assays, with retention times (R_t_) of 30.43 (1), 30.54 (2), 28.81 (3), 28.75 (4), and 27.60 min (5), respectively (Table 3). The HPLC-ESI/MS/MS analysis of each typical peak in negative ion mode for these glycosides were m/z^+^ = 417.118 (1), 447.092 (2), 463.087 (3), 463.091 (4), and 447.92 (5) (Table 3), and for their corresponding flavone aglycones were m/z^+^ = 254.653 (isoliquiritigenin), 286.081 (kaempferol), 301.932 (quercetin), 301.929 (quercetin), and 284.308 (luteolin), respectively (data not shown), signifying that these glycosides (1–5) were all monoglycosides. Then, the structures of the formed glycosides (1–5) were further elucidated from the ^1^H and ^13^C NMR spectra. As shown in Table 3, the ^1^H and ^13^C NMR spectral data of the glycoside 1 was consistent with the reported literature^23^ and identified as isoliquiritin (Isoliquiritigenin-4-*O*-glucoside), the glycoside 2 was consistent with the reported literature^24^ and identified as astragalin (Kaempferol-3-*O*-glucoside), the glycoside 3 was consistent with the reported literature^25^ and identified as hyperoside (Quercetin-3-*O*-galactoside), the glycoside 4 was consistent with the reported literature^24^ and identified as isoquercitrin (Quercetin-3-*O*-glucoside), and the glycoside 5 was consistent with the reported literature^26^ and identified as cynaroside (Luteolin-7-*O*-glucoside). The structural formulas of these glycosides were provided in Fig. 4.

Chromatographic data demonstrated that the purified *Md*P2′GT exhibited additional activities towards the chalcone (isoliquiritigenin), flavonols (kaempferol and quercetin), and the flavone (luteolin) to produce the corresponding glycosides isoliquiritin, astragaline, hyperoside, isoquercitrin, and cynaroside. Besides, the formed glycosides in seven donor/acceptor combinations were all *O*-glycosidic compounds, indicating *Md*P2′GT is an *O*-glycosyltransferase (*O*GT). Remarkably, only one hydroxy group on the glycosyl acceptor was glycosylated by *Md*P2′GT, resulting in the production of a single glycoside in each donor/acceptor combination. These unique features suggest *Md*P2′GT a special P2′GT that is not only high region specificity regarding its sugar attachment site but also accept a wide range of substrates and do not produce by-products during reactions.

### Kinetic parameters of *Md*P2′GT

The apparent kinetic parameters of *Md*P2′GT for the seven accepted substrate combinations were determined under optimal conditions to evaluate its substrate preference. As shown in Table 4, *Md*P2′GT exhibited the lowest K_m_ for UDP-glucose (7.53 μM) and phloretin (0.50 μM). The k_cat_ for UDPG and phloretin were 21.33 s^−1^ and 9.87 s^−1^, respectively, and were the highest among the seven combinations. Accordingly, the calculated catalytic efficiencies (k_cat_/K_m_) were the highest for UDPG (2.83 s^−1^ μM^−1^) and phloretin (19.73 s^−1^ μM^−1^). Besides, the highest specific activity (46.82 μkat/Kg protein) was also observed in the UDPG/phloretin combination. Thus, UDPG and phloretin were the most preferred donor/acceptor combination for *Md*P2′GT among the seven donor/acceptor combinations. Compared to reported P2′GTs, *Md*P2′GT exhibited a lower K_m_ and a higher k_cat_/K_m_ than that of UGT88F1 in the UDPG/phloretin combination, which is the lowest K_m_ value and the highest k_cat_/K_m_ reported (Table 5)^19^,^20^,^21^, demonstrating it is a novel P2′GT and favors phloridzin (2′-*O*-glycoside) synthesis the most. Besides to the 2′-*O*-glycoside (phloridzin), *Md*P2′GT also could efficiently produce 3-*O*-glycosides (isoquercitrin, hyperoside and astragaline) with k_cat_/K_m_ of 6.99 s^−1^.μM^−1^, 5.34 s^−1^.μM^−1^, and 2.70 s^−1^.μM^−1^ in the ADPG/Quercetin, UDP-Gal/Quercetin, and UDPG/Kaempferol combinations, respectively (Table 4), while it displayed relatively lower k_cat_/K_m_ in the UDPG/Isoliquiritigenin and UDPG/Luteolin combinations to produce small amount of 4-*O*-glycoside (isoliquiritin) and 7-*O*-glycoside (Cynaroside) (Table 4). These highly site-specific glycosylation characterizations and divergent product profiles favor the potential use of *Md*P2′GT as a commercial biocatalyst. Additionally, purified *Md*P2′GT exhibited optimal activity at pH values of 7.5 to 8.5 and temperatures from 30 °C to 40 °C depending on the combination of substrates. Strikingly, the specific activity and kinetic parameters of *Md*P2′GT were dramatically influenced by the sugar donor and acceptor combinations, e. g. *Md*P2′GT displayed maximal specific activity and optimal kinetic parameters in the UDPG/phloretin combination but only 10% of its maximal specific activity and poorest kinetic parameters in the ADPG/phloretin combination, while nearly 70% of the maximal specific activity was restored in the ADPG/quercetin combination. This donor-acceptor interaction is beneficial for *Md*P2′GT as a promising biocatalyst because the affinity and turnover rates toward the substrate can be adjusted by modulating the type of NDP-sugars or acceptor substrates.

### Molecular modeling and docking

**Figure 5:**
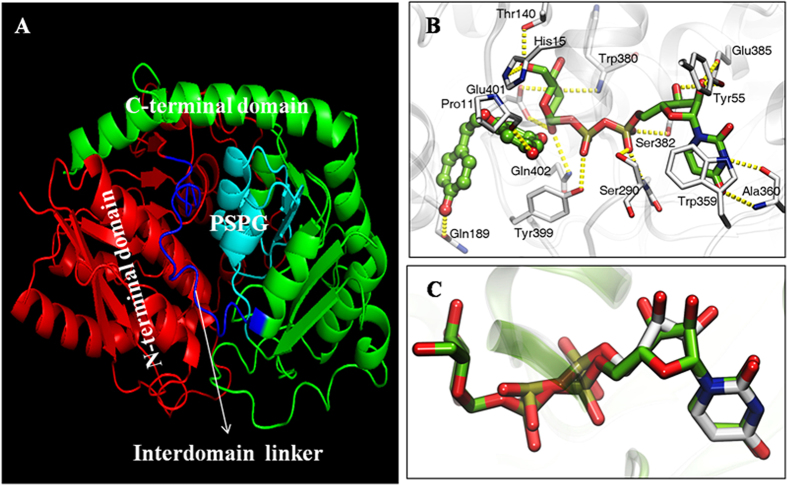
Structural analysis of *Md*P2′GT. (**A**) Ribbon diagram of the *Md*P2′GT homology-based structure with the N-terminal domain (red), C- terminal domain (green), interdomain liker region (blue), and the PSPG motif (cyan). (**B**) Overlay of the UDPG/phloretin complex with *Md*P2′GT. (**C**) Overlay of the inhibitor (UDP) with *Md*P2′GT.

Until now, there is no available crystal-based 3D structures for P2′GTs^3^,^14^,^19^,^20^,^21^. The homology-based structure for *Md*P2′GT was model using coordinates from the crystal structure of one plant GT (Accession no. **2vg8A**) which shares sequence identity with *Md*P2′GT as high as 34.07%^27^. As expected, *Md*P2′GT consisted of N- and C-terminal domains with a similar α/β/α fold (often referred to as a Rossmann fold).These two domains were connected by the interdomain linker (Fig. 5A). The “putative secondary plant glycosyltransferase (PSPG)” consensus sequence of 44 amino acids found in all Leloir GTs that is thought to be involved in binding of the activated NDP-sugar donor^9^,^19^ was observed in the C-terminal domain (Fig. 5A). We also explored molecular docking studies to elucidate the interactions between *Md*P2′GT and its preferred substrates (UDPG and phloretin) as well as its most potent product inhibitor (UDP). Five residues (W359, A360, S382, E385, and Y399) in PSPG and two residues (Y55 and S290) out of PSPG were predicted to interact with the uridine diphosphate moiety of UDPG in the form of hydrogen binds, the residues E401, Q402, W380, H15, and T140 were predicted to interact with the 2″-OH, 3″-OH, and 6″-OH on glucose residue of the UDPG in the form of hydrogen bonds (Fig. 5B). For sugar acceptor phloretin, 2′-OH group gave the best fitness score by stabilizing H-bond formation to the sugar donor, 4′- and 4-OH groups were predicted to interact with the residues P11 and Q189 in the form of H-bonds, respectively, the 6′-OH group pointed in the opposite direction from the UDPG (Fig. 5B). It is therefore not surprising that only one 2′-O-glycoside (phloridzin) was synthesized by *Md*P2′GT in UDPG/Phloretin and ADPG/Phloretin combinations. Besides, UDP, one by-product generated during the enzyme reaction, was observed to interact with the same active site residues as that with the uridine diphosphate moiety of UDPG (Fig. 5C). In this case, UDP will complete with UDPG to irreversibly bind to their active sites, leading to the reduction of *Md*P2′GT’s affinity to UDPG.

## Discussion

The predominant phenolic compound phloridzin (phloretin 2′-*O*-glucoside) in *Malus* species has various beneficial biological and pharmacological activities^3^,^11^,^12^,^19^,^20^,^21^. Compelling evidence has suggested that P2′GT is a key enzyme catalysing the rate-limiting step in the phlorizin biosynthetic pathway^12^,^21^. Recently, it has reported seven plant P2′GTs (UGT88F1, UGT88F2, UGTA15 (Accession no. **AAZ80472**), UGT71K1 (Accession no. **ACZ44835**), UGT71K2 (Accession no. **ACZ44837**), UGTA16 (Accession no. **ACZ44836**), and DicGT4 (Accession no. **BAD5200**))^14^,^19^,^20^ and one bacterial P2′GT (YijC, Accession no. **AAU40842**)^3^, but they suffer from narrow substrate range or low region selectivity. Besides, these reported P2′GTs from *Malus* display low sequence identities with each other (34.1–46.3%)^20^. Therefore, the presence of possible further phloretin glycosylating enzymes with novel characterizations cannot be excluded. In this work, a novel P2′GT (*Md*P2′GT) with highly region specificity regarding the sugar attachment site and relatively broader substrate acceptance, was purified from the generally recognized as safe (GRAS) organism engineered *P. pastoris* GS115 and then characterized.

DEAE-anion-exchange chromatography, the first use in P2′GT purification to our knowledge, effectively removed more than 50% of impurities and increased enzyme specific activity to approximately 60% via the effective adsorption, demonstrating it was a powerful tool for *Md*P2′GT purification. Besides, DEAE Sepharose is a weak anion-exchange resin showing much higher affinity and capacity for the negatively charged bio-molecules than that for the positive or neutral charged ones^28^, signifying *Md*P2′GT was a negatively charged P2′GT, in good agreement with the deduced pI of 5.8^19^,^20^. However, the molecular mass of the purified *Md*P2′GT was a little larger than the expected MW due to the low glycosylation pattern. Similar results have been observed in other GTs^29^,^30^. They reported that foreign enzyme expressed in *P. pastoris* can be hyper- or low- glycosylated when directed to secretion. This hyper- or low- glycosylation pattern can increase the MW and may affect the recombinant enzyme activity if its native structure does not contain the sugars. Fortunately, the catalytic activity of purified *Md*P2′GT was not affected by the low-glycosylated pattern, which might be explained by the glycosylation sites that positioned far away from the predicted active sites in space (Fig. 5B). Instead, the low N-glycosylated pattern is favorable because it frequently increases the half-lives of foreign proteins expressed in *P. pastoris*^30^,^31^. In addition, the simple deglycosylation treatments in the present study might facilitate the determination of favorable conditions for advanced structural research on *Md*P2′GT, as the flexibility and heterogeneity of carbohydrate moieties potentially affect the crystallization^32^,^33^.

Our data showed that purified *Md*P2′GT preferred alkaline conditions, suggesting the amino acids ionized at alkaline pH might be present in the catalytic site^34^. Similar behavior was observed in UGT88F1, UGT88F2, and YjiC, but different from UGT71A15, UGT71K1, UGT71A16, UGT71K2, and DicGT4, which have the optimal pH values at 7.7, 8.0, 8.0, 6.75, 6.75, 6.75, 6.25, and 6.0, respectively (Table 5)^3^,^14^,^19^,^20^. Moreover, the optimal and stable temperatures of purified *Md*P2′GT dramatically differed from those for reported P2′GTs (UGT88F1, UGT88F2, UGT71K1, and UGT71K2), which have optimum temperatures of 25 °C, 45 °C, 40 °C, and 25 °C, respectively (Table 5)^19^,^20^, and exhibit thermo-stability at temperatures as high as 80 °C^19^,^20^. These differences might result from the glycosylation in the amino acid sequences, the binding ability of the substrates to the enzyme active sites, the backbone structure determining the overall shape of the acceptor pocket, or the isoenzyme form and the amino acid resides under ambient conditions^9^,^31^,^35^. Other factors, such as expression vector and host strain, organisms, apple cultivars, genotype, as well as the edaphic and environmental (temperature, salinity, water stress and light intensity) conditions^1^,^3^,^14^,^20^, may also affect these properties. However, the observed enzyme inhibition by Co^2+^ and Cu^2+^ ions may be due not only to their effects on *Md*P2′GT itself but also the destruction of phloretin, since the Co^2+^, Cu^2+^ and Hg^2+^ have been reported to damage polyphenol anthocyanins that has properties similar to those of phloretin^36^,^37^. Notably, similar GT inhibition by UDP, just 5 mM was sufficient to completely inactivate *Md*P2′GT, has been reported previously and addressed by using the glycosyltransferase-catalyzed cascade reactions that utilize UDP dependent conversion of sucrose to regenerate the UDP-glucose donor with sucrose synthase^7^,^8^,^37^. The sucrose synthase reaction not only served to overcome the UDP inhibitory effect but also to solve the problem of cost-effective supply of the NDP-sugar substrate.

**Figure 6:**
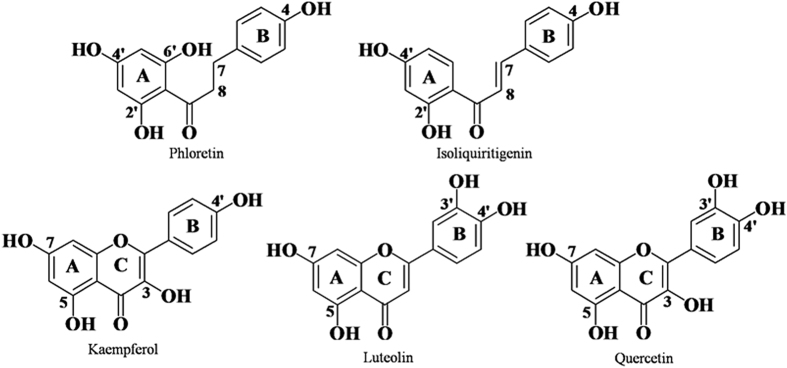
Structural formulas of the polyphenols acting as the acceptors of *Md*P2′GT.

P2′GTs are in complexes with substrates and their corresponding products, such as UGT88F1 and UGT88F2 with phloretin as substrate and strictly region-specific for the position 2′ to produce phloridzin, other reported P2′GTs with phloretin as substrate producing a mixture of mono-glycosides, di-glycosides and tri-glucosides^3^,^14^,^19^,^20^,^21^. In addition to phloretin, UGTA15, UGT71K1, and UGT71K2 also accept isoliquiritigenin, kaempferol, and quercetin as substrates^20^,^21^, and DicGT4 accept kaempferol and quercetin as substrates^9^. Another polyphenol luteolin can be utilized by UGT71A15 and UGT71A16 as well^20^. Besides to these polyphenols, the reported P2′GTs (except UGT88F1) also accept other compounds as substrates (Table 5)^14^,^20^,^38^. In present study, *Md*P2′GT could transfer the glucose moiety from the activated UDPG to phloretin specifically at the position 2′-OH to form phloridzin, which was similar to UGT88F1 and UGT88F2 but different from UGT71A15, UGT71K1, UGT71K2, UGT71A16, YijC, and DicGT4. Similar to other reported P2′GTs (except UGT88F1 and UGT88F2), *Md*P2′GT exhibited additional high activity towards isoliquiritigenin, kaempferol, quercetin, and luteolin, the formed corresponding glycosides, however, have remarkable and significant differences. E. g. phloretin and isoliquiritigenin only differ by a single C7-C8 bond and a C7-C8 double bond respectively on the flexible open-chain three-carbon linker connecting the two aromatic rings (Fig. 6)^12^,^23^. Due to this high structural similarity, 2′-OH on isoliquiritigenin should have been glycosylated in the UDPG/isoliquiritigenin combination, whereas only 4-*O*-glucoside (isoliquiritin) was detected (Fig. 4). It might be attributable to that the amount of 2′-*O*-glucoside was too low to be detected, but in scaled up reactions, it was still not detected. We speculate the presence of a C7-C8 double bond on the flexible open-chain three-carbon linker might create a steric hindrance on the 2′-OH and made the 4-OH amenable to be glycosylated based on the similar finding in previous studies^9^,^10^,^39^,^40^. Besides, isoquercitrin and hyperoside, which are native or accumulated as their aglycones in apples^41^,^42^, could be synthesized by *Md*P′2GT but couldn’t by the reported P2′GTs. Reported P2′GTs such as UGTA15, UGT71K1, UGT71K2, and DicGT4 can accept quercetin as substrate, but the sugar donor must be UDPG, and the formed glycosides were quercetin-3-*O*-glucoside (quercitrin) and 7-*O*-glucoside not isoquercitrin^14^,^20^. Furthermore, *Md*P2′GT’s affinities to UDPG and phloretin, the substrate for phloridzin synthesis^12^,^21^, are also relatively high compared to reported P2′GTs. The K_m_ of *Md*P2′GT at optimal pH 8.0 and temperature of 30 °C are 0.61% to 80.65% of the K_m_ of reported P2′GTs from other apple cultivars or organisms (Table 5)^19^,^21^. The k_cat_/K_m_ of *Md*P2′GT are 1.25-fold to three orders of magnitude to reported P2′GTs for the phloridzin synthesis (Table 5)^19^,^21^. Therefore, *Md*P2′GT shows the best enzymatic efficiency and favors phloridzin synthesis the most. Also *Md*P2′GT shows extraordinary catalysis properties for phloridzin synthesis. 370 mg/L phloridzin (850 μM) was obtained from 1.0 mM phloretin with purified *Md*P2′GT as catalyst, which was the highest among all reported phloridzin yield of 1.29 × 10^−2 ^mg/L^19^, 310 mg/L^7^, 1.18 × 10^−4 ^mg/L^21^, 5.27 mg/L^3^, 13.2 mg/L^14^. These significant differences between *Md*P2′GT and reported P2′GTs couldn’t be solely attributable to their primary sequence identity (13.2–92.6%, Table 6), which can be illustrated by the two reported plant P2′GTs named UGT88F1 and UGT88F2. These two P2′GTs both belong to the UGT 88family and share 99.0% amino acid sequence identity (Table 6)^20^ but the UGT88F2 displays higher optimal pH, temperature, and phloridzin yield as well as exhibits additional activity to hydrolyze phloridzin to phloretin (Table 5)^7^,^8^,^20^, whereas the UGT88F1 exclusively glycosylates 2′-OH on phloretin^19^. The two other UGTs designated UGT73A5 and UGT71F2 from *Dorotheanthus bellidiformis* provide an example of the contrary. These two UGTs show ~20% amino acid sequence identity but glucosylate the same set of acceptor molecules forming the same products^9^,^40^. Another two plant P2′GTs designated UGT71K1 and UGT71K2 share 93.1% amino acid sequence identity (Table 6) but they also use the same compounds as sugar donors and acceptors and produce the same glycosides^20^.

It has reported that the highly conserved secondary and tertiary structures are the key factors that influence the substrate specificity and k_cat_/K_m_ values for GTs^9^,^10^,^27^,^43^. In structural analysis, *Md*P2′GT features an α/β/α/β structure (Fig. 5A) while the reported P2′GTs adopt a β/α/β fold in PSPG motif^14^,^19^,^20^. This extra α- helix structure can create a more favorable hydrophobic environment to offer stabilizing interactions between substrates and *Md*P2′GT than reported P2′GTs. Moreover, the glycine residues in PSPG motif (Fig. S1) provide a small size of the side chains, which can confer *Md*P2′GT enough space availability to the substrates for binding the active packet. These unique structural features might not only give *Md*P2′GT a lower K_m_ toward UDPG than the reported P2′GTs but might also offer it additional activities to the other UDP-sugar donor UDP-Gal. The interdomain linker is another crucial factor determining the substrates binding due to the highly flexibility with respect to length and sequence for UGTs^9^,^10^,^27^,^40^,^44^. Both linker spans and sequences of *Md*P2′GT were the same to UGT88F1 and UGT88F2 but different from other P2′GTs^3^,^14^,^19^,^20^, and *Md*P2′GT shared high sequence identity with UGT88F1 (92.6%) and UGT88F2 (92.1%) but low with other P2′GTs (13.2–33.6%, Table 6), which might result in that ADPG could be utilized by *Md*P2′GT, UGT88F1 and UGT88F2 as another sugar donor but couldn’t by the other P2′GTs. Changes in the PSPG motif and the interdomain linker region affecting the affinity and types of sugar donor to the enzyme binding sites are also proposed for other crystallized GTs^9^,^10^,^39^,^40^. The highly divergent residues in the acceptor pocket confer GTs large differences in their individual range of acceptors^9^,^10^,^27^,^40^. For *Md*P2′GT, the functional 2′-OH group on phloretin can be positioned near to the His residue (H15) acting as the general base facilitating deprotonation of the acceptor while reported P2′GTs such as YjiC, UGT71K1 and UGT71K2 carry a Gly residue, UGTA15 and UGTA16 carry a lle residue, and DicT4 carries a large phenylalanine residue at this position. These residues pointing into the acceptor binding pocket may hamper deprotonation of the acceptor and therefore affecting the catalytic efficiency (k_cat_/K_m_). Similar findings were observed in other previous reports^9^,^10^. Besides, inter- and intradomain interactions, confer stabilization in the firm of S-S bridges, salt bridges and H-bond formation to the secondary and tertiary structure^9^,^40^, are important for activity and specify as well. Furthermore, the differences in k_cat_/K_m_ between *Md*P2′GT and reported P2′GTs may also be related to the enzyme conformation stabilized by the glycosylation procedure^33^. The usual binding of the enzyme to the substrate will not be imbedded or prevented by ambience factors after glycosylation, which is commonly reflected by an increase in k_cat_/K_m_. Similar results were observed in previous reports^31^,^35^ but the opposite results exist as well^29^,^33^. These opposite researches reported that the glycosylation of the enzymes is usually accompanied by a reduction in the substrate affinity due to the steric crowding caused by the presence of carbohydrate molecules near the substrate binding site. This discrepancy need further investigated.

Among the accepted poplyphenols, *Md*P2′GT preferred phloretin the most, followed by the kaempferol and isoliquinitigenin, and the worst one is luteolin when used UDPG as sugar donor (Table 4). Compared to phloretin, kaempferol and luteolin contain an extra C ring (Fig. 6) that may affect their binding affinity. The large aromatic residue Tyr399 in the predicted acceptor binding pocket (Fig. 5B) could create a steric hindrance to the extra C ring and locally affect the binding affinity. In contrast, there is no steric hindrance to the flexible open-chain three-carbon linker, making the functional 2-OH 2′-OH group on phloretin more amenable to be glycosylated. However, *Md*P2′GT exhibits a lower k_cat_/K_m_ toward isoliquiritigenin than kaempferol, although isoliquiritigenin has similar structure to phloretin with a flexible open-chain three-carbon linker. This discrepancy might be related to the presence of a C7-C8 double bond, lacking OH group on 6′ position of the A ring, and the absence of isoliquiritigenin in apples^41^,^42^. Luteolin lacks one OH group at the C3 position of the C ring but has one extra OH group at the C3′ position of the B ring compared to kaempferol (Fig. 6), which leads to the sugar moiety of UDPG transferred to the 7-OH group on the A ring. Also, luteolin is not natural constituents of apple fruit but kaempferol is^41^,^42^. All these differences make *Md*P2′GT exhibit a lowest k_cat_/K_m_ toward luteolin. In addition, quercetin contains one extra OH group at the C3′ position of the B ring compared to kaempferol (Fig. 6), it speculate that the residue Tyr399 can produce a stronger hindrance to quercetin than kaemferol making *Md*P2′GT exhibit a higher k_cat_/K_m_ toward kaempferol than quercetin. However, the k_cat_/K_m_ with UDPG/kaempferol combination was much lower than that with ADPG/ quercetin and UDP-Gal/ quercetin combinations for *Md*P2′GT (Table 4). On the other hand, the results signify the activated NDP-sugars may also influence the regioselectivity and catalytic efficiency of the Leloir GTs. One previous study also reports the activated NDP-sugars can influence the conformation of enzyme showing improved bioactivity^45^. This is also the first to report the donor-acceptor interactions during the final glycosylation step of phloridzin using a phloretin glycosyltransferase originating from apples.

As a novel P2′GT showing predominantly region-specific concerning the sugar attachment site and favoring phloridzin biosynthesis the most, *Md*P2′GT illustrates a distinctive set of key residues for substrates recognition. Moreover, the outstanding region-specific and broader substrate acceptance makes *Md*P2′GT an interesting enzyme for a promising catalyst in industrial preparation of phloridzin, isoquercitrin, hyperoside, and astragaline. Due to the no information on its crystal structure, the catalysis mechanism of *Md*P2′GT should be further elucidated by site directed mutagenesis or resolving its crystal structure or *Md*P2′GT-substrate complex in future study.

## Methods

### Chemicals

NEM and PHMB with purity ≥98% were purchased from the Aladdin Reagent Co. (Shanghai, China). Marker proteins, PNGase F, Endo H, nucleoside diphospate (NDP)-sugars, AEBSF, Yeast Nitrogen Base (YNB, molecular biology grade), biotin and Geneticin (G418) with purity ≥98% were purchased from Sigma-Aldrich (St. Louis, MO, USA). The glycosyl acceptors evaluated in this study, 3-hydroxyphloridzin and trilobatin, were purchased from Chengdu Must Bio-Technology Co., Ltd. (Chengdu, China) with purity >98%. All other reagents were of analytical grade.

### *Md*P2′GT activity

The *Md*P2′GT activity assay was performed following a previous method with minor modifications^19^. Reaction mixtures consisted of 0.5 ml buffer A (50 mM Tris/HCl, pH 7.5, and 5 mM DTT), 270 μM UDPG, 20 μM phloretin (dissolved in DMSO) and 20 μg purified *Md*P2′GT. After incubation at 30 °C for 30 min, the reactions were immediately terminated by freezing in liquid N_2_ and lyophilized. Methanol (0.25 ml) was then added to dissolve the components, and the product phloridzin was quantified by the HPLC-DAD method on a WondaSil^®^ C_18_ column (4.6 × 250 mm, ID = 5 μm, Shimadzu, Kyoto, Japan) as described in our previous study^42^. One unit of *Md*P2′GT activity was defined as the amount of enzyme needed to produce 1 μmol product per second (s) at 30 °C and pH 7.5, and specific activity was expressed as μkat/Kg protein.

### Expression and purification of *Md*P2′GT

The open reading frame of *Md*P2′GT was sub-cloned into the *Sna*BI and *Not*I sites of pPIC9K (Novagen) and transformed into *P. pastoris* GS115 (Novagen) by electroporation^46^,^47^. Engineered *P. pastoris* GS115 carrying pPIC9K/*Md*P2′GT was cultured in a 5 L bioreactor (Applikon^®^ Biotechnology, Foster City, USA) with a working volume of 1 L buffered minimal sorbitol medium (BMSM, 100 mM potassium phosphate, pH 7.0, 1.34% YNB, 4 × 10^−5^% biotin, and 5.0% sorbitol) in the presence of G418 (4.0 mg/ml) overnight at 28 °C, 280 rpm, and were induced, at the optical density (OD_600_) of 10.0–15.0, with 0.75% methanol at 25 °C for 122h^46^,^47^. The resulting supernatant was harvested, dialyzed and lyophilized to a powder. The powder was dissolved in buffer B (50 mM Tris/HCl, pH 7.5, 2 mM MgCl_2_, 5 mM DTT, 0.5 mM AEBSF, and 5% glycerol), filtered through a 0.22-μm membrane (Millipore) and then loaded onto a pre-equilibrated diethyl-aminoethanol (DEAE) Sepharose column (HiTrap-DEAE-FF, 1 ml, Amersham Biosciences) operated with an AKTA purifier system (Amersham Biosciences, Uppsala, Sweden)^28^. The column was first washed with buffer B for 5 column volumes (CVs) and then eluted with buffer B containing NaCl (500 mM) a linear gradient (0 to 0.5 M) at a flow rate of 1.0 ml/min. The fractions of interest (identified by activity towards UDPG and Phloretin) were collected and dialyzed against buffer C (50 mM Tris/HCl, pH 7.5, 200 mM NaCl, 2 mM MgCl_2_, 5 mM DTT, and 0.5 mM AEBSF) at 4 °C. The dialyzed solution was further purified under native conditions on a gravity flow column (10 ml, GE Healthcare) containing 1 ml cOmplete His-Tag Purification Resin (Roche CH 05-893-682-001) pre-equilibrated with buffer C^48^. The column was stepwise washed with 20 CVs of 40–500 mM imidazole in buffer C. The fractions of interest, judged by SDS-PAGE and enzyme activity assays, were applied to the gel filtration on a Superdex 75 10/300 GL column (24 ml, Amersham Biosciences) using buffer C as the eluent^49^. The final purified fractions were pooled, desalted, and concentrated using a 10 K membrane ultracentrifugation system (Ultrace-10, Millipore, Massachusetts, USA)^49^. The enzyme purity was estimated by specific activity as described in enzyme activity assay, Coomassie Blue R-250 (Bio-Rad, CA, USA) stained 8% SDS-PAGE, and Western blot with an anti-His antibody^19^, respectively. The total protein was determined by the Bradford method using a Bradford kit with bovine serum albumin as the standard^47^,^48^.

### Molecular weight evaluation

The concentration of the purified *Md*P2′GT solution was adjusted to 5 mg/ml and loaded onto a Sephacryl S-300 column (GE Healthcare, Piscataway, USA; 1.6 × 60 cm) pre-equilibrated with buffer D (50 mM Tris-HCl, pH 7.5, 200 mM NaCl, and 5 mM DTT) for SEC^31^. Five marker proteins (75 kDa conalbumin, 67 kDa bovine serum albumin, 45 kDa egg albumins, 29 kDa carbonic anhydrase, and 14.9 kDa lysozyme) were used to calibrate the column and generate a standard curve based on the partition coefficient of K_av_ (abscissa) and lgMr (ordinate). K_av_ is calculated on the the molecular mass and the elution volume of each protein; Mr is the molecular mass of the corresponding marker proteins.

### Glycosylation analysis

Glycosylation analysis was performed by enzyme treatment with PNGase F and Endo H according to the methods described by Luciana Facchinetti *et al*.^31^ and Schlenzig *et al*.^35^. The sizes of the deglycosylated *Md*P2′GT, PNGase F and Endo H were analyzed by SDS-PAGE and SEC as described in the molecular weight analysis. The native and deglycosylated *Md*P2′GT bands in the SDS-PAGE gel were excised and analyzed by matrix-assisted laser desorption ionization-time of flight mass spectrometry (MALDI-TOF/MS) in the linear mode as described by Schlenzig *et al*.^35^.

### Optimum pH and temperature and stability

The optimal pH was assessed in the pH range of 4.0–10.0 using 100 mM potassium phosphate buffer (pH 4.0–7.0) and Tris/HCl buffer (pH 7.0–10.0) at 30 °C. The optimal temperature was determined in the temperature range of 15–75 °C at pH 8.0. For the pH stability evaluation, *Md*P2′GT was incubated at 30 °C for 24 h at pH values varying from 6.5 to 9.5. For the temperature stability assessment, *Md*P2′GT was incubated at pH 8.0 at several temperatures (25, 35, 45, 55, and 65 °C) for different periods of time (0, 10, 20, 30, 40, 50, and 60 min). The residual activity of *Md*P2′GT was determined as described in the enzyme activity assay section and expressed as the relative activity compared with the initial activity, which was considered 100%.

### Effects of metal ions and other compounds

The effects of metal ions (monovalent, divalent, and trivalent metal ions), chelating agent (EDTA), protectants (glycerol and AEBSF), thiol-containing compounds (β-ME and DTT), thiol inhibitors (PHMB and NEM), substrate co-solvents (methanol and DMSO), substrate analogs (UDP, uridine triphosphate (UTP), uridine monophosphate (UMP), and uridine) and product analogs (trilobatin and 3-hydroxyphloridzin) on the activity of purified *Md*P2′GT were examined as described in the enzyme activity assay section. The inhibition effects were calculated and expressed as relative activity (100%). The half-maximal inhibitory concentration (IC_50_) values, defined as the concentration of inhibitor required to reduce the original activity of *Md*P2′GT activity by 50%, were used to evaluate the effects.

### Substrate specificity assays

Nine potential sugar donors, including UDPG, ADPG, guanosine diphosphate glucose, cytidine diphosphate glucose, thymidine diphosphate glucose, UDP-Gal, UDP-xylose, UDP-rhamnose, and UDP-pentose, and five types of potential sugar acceptors, including hydroxycinnamic acids (chlorogenic acid, caffeic acid, 4-coumaric acid, 3-coumaric acid, and 2-coumaric acid), chalcones (phloretin, isoliquiritigenin, naringenin chalcone, eriodictyol chalcone, and butein), flavanols (catechin, epicatechin, epigallocatechin, epicatechin gallate, and epigallocatechin gallate), flavonols (quercetin, dihydroquercetin, rutin, kaempferol, and myricetin), and flavones (baicalein, luteolin, eriodictyol, chrysin, and apigenin) were evaluated as potential *Md*P2′GT substrates. For chromatographic identification of the enzyme products, substrate specificity assays were scaled up to a total volume of 20 ml containing approximately 1.0 mg of purified *Md*P2′GT, 1.0 mM acceptor substrate, and 10.8 mM donor substrate in buffer A. The reactions were performed at 30 °C and pH 7.5 for 15 h and terminated by adding 50 μl of glacial acetic acid. After lyophilization in liquid N_2_, the powder containing the enzyme products was re-dissolved in 10 ml of methanol and then filtered through a 0.22-μm membrane (Millipore). The formed glycosides were first evaluated using the same HPLC-DAD method as described in our previous study^42^. Then, the target HPLC peaks were subjected to LC-MS/MS (Foster City, CA, USA) on a 4000 QTrap system from Applied Biosystems equipped with an electrospray ionization (ESI) interface and a HPLC system (Shimadzu, Tokyo, Japan)^50^. The chemical structures of target glycosides were further confirmed by NMR assays in DMSO-*d*_6_ solvent (Sigma-Aldrich, St. Louis, USA)^24^. ^1^H NMR and ^13^C NMR spectroscopic analysis was performed on a Bruker Avance 500 MHz spectrometer (Bruker Corp., Billerica, USA) with. Chemical shifts were expressed in δ ppm with coupling constants (Ј) determined using tetramethylsilane (TMS) as an internal standard.

### Kinetic parameters

The kinetic parameters of *Md*P2′GT were determined by measuring its activity in the presence of various concentrations of sugar donor and acceptor substrates. For the donor substrates, the acceptor substrates concentration was fixed at 1.0 mM, with donors concentrations varying from 27 μM to 25.4 mM. For the acceptor substrates, the donor substrates concentration was fixed at 10.8 mM, with acceptor concentration varying from 4.0 μM to 2.0 mM. Kinetic constants (K_m_, V_max_, K_cat_) were calculated from Lineweaver-Burk plots. All assays were performed in triplicate.

### Molecular modeling and docking

The 3D structures of *Md*P2′GT was modeled by submitting its full-length amino acid sequences (Fig. S1) to the I-TASSER ( http://zhanglab.ccmb.med.umich.edu/). Structures of the substrates and inhibitor were built by ChemBioDraw Ultra 12.0 and their ground state conformation predicted was docked into the possible catalytic pocket of the appropriate *Md*P2′GT model according to the previous report^10^,^27^.

## Additional Information

**How to cite this article**: Zhang, T. *et al*. Purification and characterization of a novel phloretin-2′-*O*-glycosyltransferase favoring phloridzin biosynthesis. *Sci. Rep.* **6**, 35274; doi: 10.1038/srep35274 (2016).

## Supplementary Material

Supplementary Information

## Figures and Tables

**Table 1:** Purification and activity of *Md*P2′GT from the bioengineered *P. pastoris* GS115 supernatant.

Purification step	Total protein (mg)	Total activity (nkat)	Specific activity (μkat/Kg protein)	^b^Purification (Fold)	Yield (100%)
Crude	1240.71 ± 50.46	0.14 ± 0.01	0.11 ± 0.01	1	1
^a^DEAE Sepharose Fast Flow	618.49 ± 31.73	4.01 ± 0. 18	6.48 ± 0.39	59	49.85
Ni^2+^ affinity (80 mM imidazole)	162.85 ± 18.96	5.40 ± 0.34	33.13 ± 1.23	301	13.13
Gel filtration (Superdex 75)	111.89 ± 8.93	4.53 ± 0.37	40.45 ± 2.01	368	9.02

^a^Determined data represents the pooled fractions of F2, F3, and F4 that displayed the highest specific activity in Fig. 1A.

^b^Calculated according to the mean of the specific activity and expressed as an integer.

**Table 2:** Effects of various reagents on purified *Md*P2′GT activity.

Addition	Concentration/(mM)	Relative activity/(100%)	IC_50_/(mM)
None	—	100	—
NaCl	500	100 ± 1.00	ND
KCl	5	99.58 ± 2.31	ND
MgCl_2_	2	130.35 ± 3.53	Activator
NiCl_2_	5	98.26 ± 2.08	ND
CoCl_2_	5	0.00 ± 0.00	0.05 ± 0.01
CuCl_2_	5	0.00 ± 0.00	0.01 ± 0.01
CaCl_2_	5	54.21 ± 1.05	5.50 ± 0.12
BaCl_2_	5	99.03 ± 2.54	ND
MnSO_4_	5	47.41 ± 1.13	4.50 ± 0.11
FeSO_4_	5	119.43 ± 3.61	Activator
AlCl_3_	5	67.81 ± 1.03	8.00 ± 0.50
FeCl_3_	5	60.78 ± 1.01	6.50 ± 0.23
EDTA	5	99.16 ± 2.21	ND
Glycerol	686	98.13 ± 2.15	ND
AEBSF	0.5	99.51 ± 2.36	ND
β-mercaptoethanol	5	99.02 ± 1.47	ND
Dithiothreitol	5	99.15 ± 1.43	ND
Methanol	1236	99.23 ± 1.41	ND
Dimethyl sulfoxide	704	99.54 ± 1.40	ND
*p*-hydroxymercuribenzoate	1	18.45 ± 1.02	0.24 ± 0.07
*N*-Ethylmaleimide	1	20.53 ± 1.08	0.28 ± 0.01
UDP	5	0 ± 0.00	0.10 ± 0.01
UTP	5	3.05 ± 0.26	2.5 ± 0.10
UMP	5	38.25 ± 1.07	4.00 ± 0.13
Uridine	5	98.17 ± 2.12	ND
Trilobatin	5	21.70 ± 1.15	3.00 ± 0.21
3- hydroxyphloridzin	5	15.65 ± 0.58	2.50 ± 0.20

Relative activities were calculated by comparison to a control in which none is added.

No effect of addition on MdP2′GT activity was defined as relative activities ≥95%.

ND: no effects detected at selected concentrations.

Activator: additions increased MdP2′GT activity at selected concentrations.

**Table 3:** Analytical data of formed glycosides in the other five donor/acceptor combinations with purified *Md*P2′GT.

Donor/acceptor combinations	Glycosides	R_t_ (min)	m/z [M-H^+^]	NMR (500 MHz, DMSO-*d*_6_)	
				δ^1^H (ppm)	δ^13^C (ppm)
UDPG/Isoliquiritigenin	1	30.43	417.118	8.009 (1H, d, J = 8.8 Hz, H-6′), 7.837 (1H, d, J = 15.5 Hz, H-8), 7.806 (2H, d, J = 8.0, H-2,6), 7.720 (1H, d, J = 15.3 Hz, H-7), 7.182 (2H, d, J = 8.7 Hz, H-3,5), 6.438 (1H, dd, J = 2.0, 8.5 Hz, H-5′), 6.314 (1H, d, J = 2.0 Hz, H-3′), 5.011 (1H, d, J = 7.2 Hz, H-1″), 4.013 (1H, dd, J = 2.0, 11.8 Hz, H-6″), 3.745 (1H, dd, J = 5.3, 11.9 Hz, H-6″)	191.97 (C-9), 166.19 (C-4′), 165.16 (C-2′), 159.66 (C-4), 143.43 (C-8), 132.10 (C-6′), 130.02 (C-2,6), 129.13 (C-1), 118.65 (C-7), 116.60 (C-3,5), 113.28 (C-1′), 107.85 (C-5′), 102.43 (C-3′), 100.41 (C-1″), 76.86 (C-3″), 76.55 (C-5″), 73.44 (C-2″), 69.90 (C-4″), 61.07 (C-6″)
UDPG/Kaempferol	2	30.54	447.092	7.949 (2H, d, J = 9.0 Hz, H-2′, 6′), 6.798 (2H, d, J = 8.5 Hz, H-3′, 5′), 6.295 (1H, d, J = 2.0 Hz, H-8), 6.110 (1H, d, J = 2.0 Hz, H-6), 5.113 (1H, d, J = 7.0 Hz, H-1″), 3.088-3.573 (6H, m, sugar protons)	178.15 (C-4), 164.62 (C-7), 161.67 (C-5), 160.15 (C-4′), 157.81 (C-9), 157.14 (C-2), 134.18 (C-3), 130.87 (C-2′, 6′), 121.49 (C-1′), 114.71 (C-3′, 5′), 104.40 (C-10), 102.97 (C-1″), 98.58 (C-6), 93.43 (C-8), 76.97 (C-5″), 76.72 (C-3″), 74.38 (C-2″), 70.09 (C-4″), 61.37 (C-6″)
ADPG/Quercetin	3	28.81	463.087	7.502 (1H, dd, J = 7.5, 2.5 Hz, H-6′), 7.497 (1H, d, J = 2.4 Hz, H-2′), 6.774 (1H, d, J = 7.0 Hz, H-5′), 6.326 (1H, d, J = 2.1 Hz, H-8), 6.120 (1H, d, J = 2.0 Hz, H-6), 5.194 (1H, d, J = 7.4 Hz, H-1″)	177.97 (C-4), 164.46 (C-7), 161.49 (C-5), 157.09 (C-2), 156.89 (C-9), 148.46 (C-4′), 144.46 (C-3′), 134.15 (C-3), 121.60 (C-6′), 121.46 (C-1′), 116.35 (C-5′), 114.88 (C-2′), 104.20 (C-10), 103.40 (C-1″), 98.51 (C-6), 93.38 (C-8), 75.80 (C-5″), 73.55 (C-3″), 71.64 (C-2″), 68.48 (C-4″), 60.47 (C-6″)
UDP-Gal/Quercetin	4	28.75	463.091	7.491 (2H, dd, J = 7.8, 2.0 Hz, H-2′,6′), 6.773 (1H, d, J = 7.8 Hz, H-5′), 6.278 (1H, d, J = 2.0 Hz, H-8), 6.091 (1H, d, J = 2.0 Hz, H-6), 5.271 (1H, d, J = 7.3 Hz, H-1″), 3.706-3.285 (6H, m, sugar protons)	177.61 (C-4), 164.17 (C-7), 161.17 (C-5), 157.13 (C-2), 156.58 (C-9), 147.98 (C-4′), 144.03 (C-3′), 133.75 (C-3), 121.34 (C-6′), 121.20 (C-1′), 115.71 (C-5′), 114.13 (C-2′), 103.81 (C-10), 102.46 (C-1″), 98.03 (C-6), 92.85 (C-8), 76.52 (C-5″), 76.25 (C-3″), 73.87 (C-2″), 69.34 (C-4″), 60.68 (C-6″)
UDPG/Luteolin	5	27.60	447.092	7.41 (1H, d, J = 2.4 Hz, H-2′), 7.35 (1H, dd, J = 8.4, 2.4 Hz, H-6′), 6.89 (1H, d, J = 8.4 Hz, H-5′), 6.77 (1H, d, J = 2.4 Hz, H-8), 6.75 (1H, s, H-3), 6.43 (1H, d, J = 2.4 Hz, H-6), 5.07 (1H, d, J = 7.2 Hz, H-1″)	181.68 (C-4), 164.34 (C-2), 162.60 (C-7), 160.72 (C-5), 156.75 (C-9), 149.10 (C-4′), 145.05 (C-3′), 121.37 (C-1′), 118.53 (C-6′), 115.07 (C-2′), 112.50 (C-5′), 105.01 (C-10), 102.47 (C-3), 99.59 (C-6), 99.01 (C-1″), 94.10 (C-8), 76.47 (C-3″), 75.81 (C-5″), 72.68 (C-2″), 69.14 (C-4″), 60.29 (C-6″)
^a^UDPG/Phloretin^a^ADPG/Phloretin	6	28.04	437.256	2.79 (2H, t, J = 7.4 Hz, H-7), 3.03 (2H, dd, J = 11.4 Hz, H-2″, 4″), 3.11 (1H, m, J = 5.1 Hz, H-5′′), 3.20 (1H, m, J = 4.7 Hz, H-3″), 3.38 (2H, m, J = 8.3 Hz, H-8), 3.62 (2H, dd, J = 5.4, 11.8 Hz, H-6″), 4.94 (1H, d, J = 6.8 Hz, H-1″), 5.86 (2H, d, J = 1.9 Hz, H-3, 5), 6.08 (1H, d, J = 1.8 Hz, H-3′), 6.59 (2H, d, J = 8.1 Hz, H-2, 6), 6.95 (1H, d, J = 8.4 Hz, H-5′)	29.43 (C-7), 45.56 (C-8), 61.02 (C-6″), 69.68 (C-4″), 73.30 (C-2″), 77.00 (C-5″), 77.08 (C-3″), 94.05 (C-5′), 96.96 (C-3′), 100.66 (C-1″), 105.37 (C-1′), 114.68 (C-2 and C-6), 128.99 (C-3 and C-5), 132.48 (C-1), 154.97 (C-4), 160.91 (C-4′), 164.58 (C-6′), 166.16 (C-2′), 205.14 (C-9)

^a^The formed glycoside in UDPG/Phloretin and ADPG/Phloretin combinations was determined in our previous unpunished study and identified as phloridzin.

**Table 4:** Enzyme characters and kinetic parameters of purified *Md*P2′GT.

Donor/acceptor combinations	Acceptor class	Products	Donors			Acceptors			Specific activity (μkat/Kg protein)	pH optimum	Temperature optimum (°C)
			K_m_ (μM)	k_cat_ (s^−1^)	k_cat_/K_m_ (s^−1^. μM^−1^)	K_m_ (μM)	k_cat_ (s^−1^)	k_cat_/K_m_ (s^−1^. μM^−1^)			
UDPG/Phloretin	Dihydrochalcone	Phloridzin	7.53 ± 1.35	21.33 ± 0.68	2.83	0.50 ± 0.02	9.87 ± 0.89	19.73	46.82 ± 3.45	8.0	30
ADPG/Phloretin	Dihydrochalcone	Phloridzin	58.67 ± 5.63	4.65 ± 0.14	0.08	4.09 ± 0.14	2.95 ± 0.10	0.72	4.90 ± 0.98	8.0	30
UDPG/Isoliquiritigenin	Chalcone	Isoliquiritin	36.19 ± 4.09	8.68 ± 0.26	0.24	2.40 ± 0.08	4.35 ± 0.15	1.81	10.37 ± 1.56	7.5	40
UDPG/Kaempferol	Flavonol	Astragaline	30.43 ± 3.11	10.83 ± 0.47	0.36	2.09 ± 0.12	5.65 ± 0.65	2.70	17.13 ± 1.43	8.5	35
ADPG/Quercetin	Flavonol	Isoquercitrin	14.57 ± 2.45	15.22 ± 0.56	1.04	1.06 ± 0.08	7.41 ± 0.70	6.99	32.18 ± 2.95	8.5	35
UDP-Gal/Quercetin	Flavonol	Hyperoside	26.19 ± 3.23	13.18 ± 0.52	0.50	1.81 ± 0.10	6.18 ± 0.23	5.24	23.13 ± 2.03	8.0	35
UDPG/Luteolin	Flavone	Cynaroside	42.59 ± 4.81	5.95 ± 0.09	0.14	2.89 ± 0.18	3.24 ± 0.18	1.12	7.80 ± 1.13	7.5	40

**Table 5:** Enzyme characters and Kinetic parameters of *Md*P2′GT and reported P2′GTs.

Enzyme	^a^ K_m_ (μM)	^a^ k_cat_ (s^−1^)	^a^ k_cat_/K_m_ (s^−1^. μM^−1^)	^a^ Vmax (mmol/s. kg protein)	^a^ Optimal pH	^a^ Optimal temperature (°C)	Substrate specificity (Donor/acceptor combinations)	Organisms	References
*Md*P2′GT	0.50	9.87	19.74	167.21	8.0	30	UDPG/Phloretin, ADPG/Phloretin, UDPG/Isoliquiritigenin, UDPG/Kaempferol, ADPG/Quercetin, UDP-Gal/ Quercetin, and UDPG/Luteolin	*M*. x *domestic a cv*. Golden delicious	This study
UGT88F1	0.62	9.72^c^	15.68	164.75^b^	7.7	25	UDPG/Phloretin, UDP-galactose/ Phloretin	*M*. x *domestica cv*. Royal Gala	19
UGT88F2	—	—	—	—	8.0	45	UDPG/Phloretin, UDP/Phloridzin	*P. communis cv.* Abbé Fetel	7, 8, 20
UGT71A15	82	0.42^d^	0.005	8 × 10^−3^	6.75	30	UDPG/Phloretin, UDPG/Naringenin chalcone, UDPG/Eriodictyol chalcone, UDPG/Isoliquiritigenin, UDPG/Butein, UDPG/Naringenin, UDPG/Eriodictyol, UDPG/Luteolin, UDPG/Kaempferol, and UDPG/Quercetin	*M*. x *domestica cv*. Rebella	20, 21
UGT71K1	—	—	—	—	6.75	40	UDPG/Phloretin, UDPG/Kaempferol, UDPG/Quercetin, UDPG/Isoliquiritigenin, UDPG/Butein	*M*. x *domestica cv*. Rebella	20
UGT71K2	—	—	—	—	6.25	25	UDPG/Phloretin, UDPG/Kaempferol, UDPG/Quercetin, UDPG/Isoliquiritigenin, UDPG/Butein	*P. communis cv.* Abbé Fetel	20
UGT71A16	—	—	—	—	6.75	30	UDPG/Phloretin, UDPG/Eriodictyol, UDPG/Apigenin, UDPG/Luteolin, and UDPG/Caffeic acid	*P. communis cv.* Abbé Fetel	20
^e^YjiC	—	—	—	—	8.0	30	UDPG/Phloretin, UDPG/geldanamycin analogs	*Bacillus licheniformis*	3, 38
DicGT4	—	—	—	—	6.0	30	UDPG/Phloretin, UDPG/Naringenin, UDPG/Kaempferol, UDPG/Quercetin, UDPG/apigenin, and UDPG/cyanidin	*Dianthus caryophyllus*	14

^a^Data was determined for the glycosylation of 2′-OH group on phloretin using the UDPG/Phloretin combination as substrate.

^b^Calculated according to the turnover rate of 9.72 × 10^−4 ^mol.s^−1^.mol^−1^ and MW = 59 kDa.

^c^Calculated according to K_m_ and MW = 59 kDa.

^d^Calculated according to K_m_ and MW = 52 kDa.

^e^Geldanamycin analogs act as the acceptors of YjiC included 18-dehydroxy-17-demethoxyreblastatin, 18-dehydroxy-17-O-demethylreblastatin, 18-dehydroxy-17-O-demethyl-4,5-dehydroreblastatin, 17-demethoxy-reblastatin, and 7-demethoxy-15-hydroxylreblastatin.

**Table 6:** Amino acid sequence identity (I) and similarity (S) of *Md*P2′GT and other reported P2′GTs.

Enzyme	UGT88F1	UGT88F2	UGT71A15	UGT71K1	UGT71K2	UGT71A16	YjiC	DicGT4	References
UGT88F2	**I: 99.0% S: 99.4%**								19, 20
UGT71A15	*I: 34.6% S: 51.0%*	I: 34.3% S: 50.9%							19, 20, 21
UGT71K1	*I: 36.1% S: 53.6%*	I: 36.1% S: 53.6%	*I: 46.3% S: 67.4%*						19, 20, 21
UGT71K2	I: 35.8% S: 53.0%	*I: 35.8% S: 53.0%*	I: 45.8% S: 67.0%	**I: 93.1% S: 95.4%**					19, 20, 21
UGT71A16	I: 34.4% S: 51.0%	*I: 34.1% S: 53.9%*	**I: 91.7% S: 96.0%**	I: 45.1% S: 66.6%	*I: 44.3% S: 65.6%*				19, 20, 21
^a^YjiC	I: 14.2% S: 46.7%	I: 14.2% S: 46.7%	I: 16.8% S: 44.4%	I: 15.7% S: 45.2%	I: 15.7% S: 45.2%	I: 16.4% S: 45.5%			3, 19, 20, 21
^b^DicGT4	I: 27.1% S: 42.3%	I: 27.3% S: 42.3%	I: 26.5% S: 41.4%	I: 27.0% S: 39.1%	I: 26.8% S: 39.4%	I: 26.5% S: 43.1%	I: 20.2% S: 48.2%		3, 14, 19, 20, 21
^c^*Md*P2′GT	***I: 92.6% S: 96.1%***	**I: 92.1% S: 95.6%**	*I: 31.6% S: 35.6%*	*I: 32.6% S: 34.6%*	I: 33.6% S: 35.0%	I: 31.8% S: 37.1%	I: 13.2% S: 26.6%	I: 27.1% S: 33.1%	This study

Values for orthologous sequences are highlighted in bold; those for paralogous sequences are in italics.

^a^Calculated according to the sequences of YjiC following the method described as Gosch *et al*.^20^.

^b^Calculated according to the sequences of DicGT4 following the method described as Gosch *et al*.^20^.

^c^Calculated according to the sequences of *Md*P2′GT shown in Fig. S1 following the method described as Gosch *et al*.^20^.
