# Supplementary material for: Purification and characterization of a novel phloretin-2′-O-glycosyltransferase favoring phloridzin biosynthesis
Source: Sci Rep. 2016 Oct 12;6:35274. doi: 10.1038/srep35274 (PMC5059724; doi:10.1038/srep35274)
Supplement: Supplementary Information [file srep35274-s1.pdf]

Supplementary Information

Purification and characterization of a novel phloretin-2'-*O*-glycosyltransferase favoring phloridzin biosynthesis

Tingjing Zhang[a], Jianqiang Liang[b], Panxue Wang[c], Ying Xu[d], Yutang Wang[a], Xinyuan Wei[a], Mingtao Fan[a]*

[a] College of Food Science and Engineering, Northwest A&F University, Yang ling, Shaanxi, 712100, China

[b] College of Life Sciences, Northwest A&F University, Yangling, Shaanxi, 712100, China

[c] Department of Food Science, University of Massachusetts, Amherst, MA01003, USA

[d] College of Life Science and Engineering, Shaanxi University of Science & Technology, Xi'an, Shaanxi, 710021, China

* Corresponding author:

Mingtao Fan, College of Food Science and Engineering, Northwest A&F University, Yang ling, Shaanxi, 712100, China

Tel: +8613892877726

Fax: +8602987092486

E-mail: fanmt@nwsuaf.edu.cn (M. Fan)

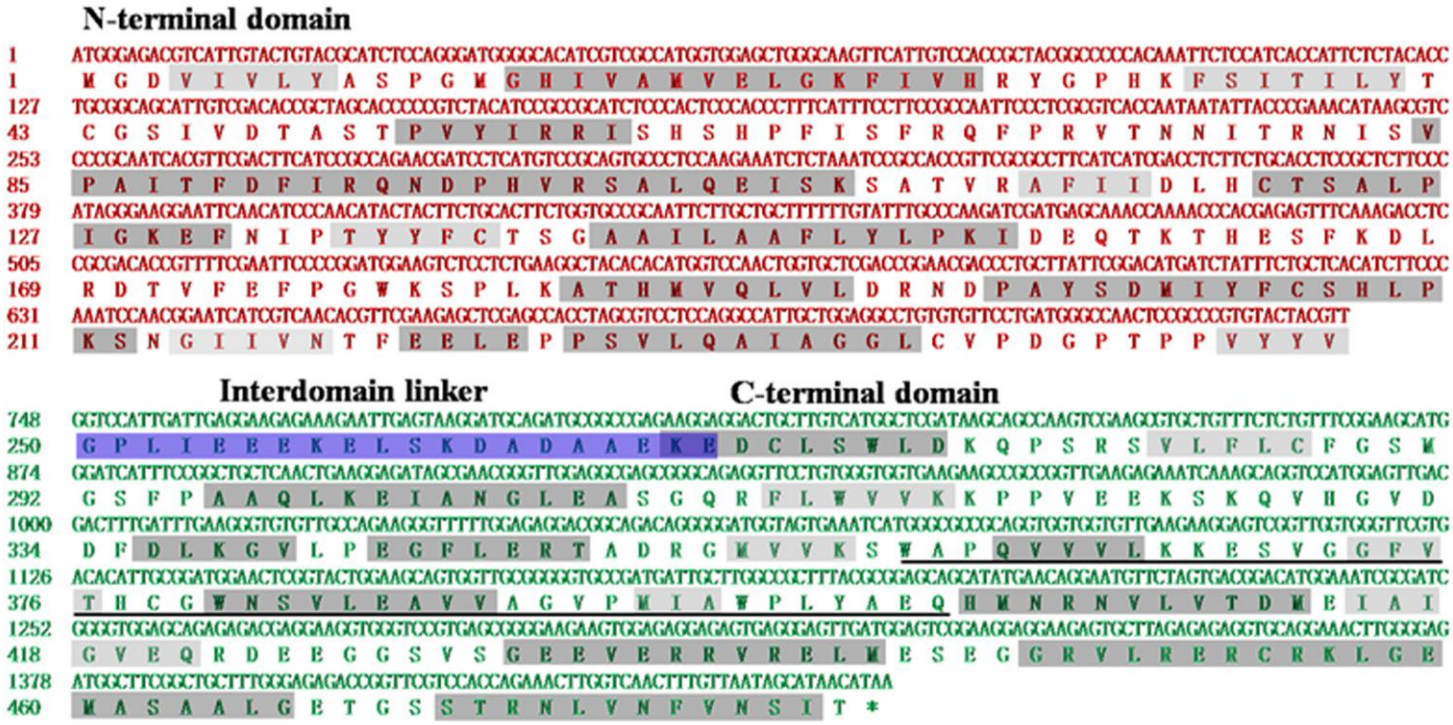

Fig. S1 Analysis of the nucleotide and amino acid sequences of *Md*P2'GT

The nucleotide sequence was indicated on the top line and the deduced amino acid sequence was shown in single letters designated below the nucleotide sequence on the second line. The residues forming the N-terminal domain were highlighted in red, residues in the C-terminal domain were highlighted in green, and residues in the interdomain linker were highlighted in blue. The residues were predicted to form the PSPG motif were marked with underline, α-helices were highlighted in dark grey, and β-strands were highlighted in light grey.
